# Applications of Mesenchymal Stem Cells in Skin Regeneration and Rejuvenation

**DOI:** 10.3390/ijms22052410

**Published:** 2021-02-27

**Authors:** Hantae Jo, Sofia Brito, Byeong Mun Kwak, Sangkyu Park, Mi-Gi Lee, Bum-Ho Bin

**Affiliations:** 1 Department of Applied Biotechnology, Ajou University, Suwon 16499, Korea; jesuswh1@gmail.com (H.J.); sofia@ajou.ac.kr (S.B.); 2 Department of Meridian and Acupoint, College of Korean Medicine, Semyung University, Chungbuk 27136, Korea; bmkwak@semyung.ac.kr; 3 School of Cosmetic Science and Beauty Biotechnology, Semyung University, 65 Semyung-ro, Jecheon-si, Chungcheongbuk-do 27136, Korea; 4 Department of Biological Sciences, Ajou University, Suwon 16499, Korea; 5 Bio-Center, Gyeonggido Business and Science Accelerator, Suwon 16229, Korea

**Keywords:** mesenchymal stem cells, skin regeneration, wound healing, skin rejuvenation, antiaging, induced pluripotent stem cell

## Abstract

Mesenchymal stem cells (MSCs) are multipotent stem cells derived from adult stem cells. Primary MSCs can be obtained from diverse sources, including bone marrow, adipose tissue, and umbilical cord blood. Recently, MSCs have been recognized as therapeutic agents for skin regeneration and rejuvenation. The skin can be damaged by wounds, caused by cutting or breaking of the tissue, and burns. Moreover, skin aging is a process that occurs naturally but can be worsened by environmental pollution, exposure to ultraviolet radiation, alcohol consumption, tobacco use, and undernourishment. MSCs have healing capacities that can be applied in damaged and aged skin. In skin regeneration, MSCs increase cell proliferation and neovascularization, and decrease inflammation in skin injury lesions. In skin rejuvenation, MSCs lead to production of collagen and elastic fibers, inhibition of metalloproteinase activation, and promote protection from ultraviolet radiation-induced senescence. In this review, we focus on how MSCs and MSC-derived molecules improve diseased and aged skin. Additionally, we emphasize that induced pluripotent stem cell (iPSC)-derived MSCs are potentially advanced MSCs, which are suitable for cell therapy.

## 1. Mesenchymal Stem Cells

Mesenchymal stem cells (MSCs) were observed for the first time in bone marrow by Cohnheim in 1867, who discovered that these cells could be the source of fibroblasts involved in wound repair [1]. Later, MSCs were first isolated and cultured in 1968 by A. J. Friedenstein. Using cells prevenient from murine bone marrow, Friedenstein observed that transplanting cell colonies to semi-syngeneic animals could originate fibrous tissue, bone and bone containing bone marrow. However, only years after, it became clear that the works made by Friedenstein were due to cells with multipotent ability. The term “mesenchymal stem cells” was presented by Caplan in 1991, after his studies with human bone marrow research [2,3]. Since then, owing to their easy isolation, expansion, and multipotentiality, MSCs have been rapidly popularized as a promising therapeutic agent for regenerative medicine. To date, it is a hot topic of research that is being explored for multiple purposes. The International Society for Cellular Therapy (ISCT) has suggested at least three conditions that can characterize MSCs. First, MSCs must adhere to a plastic culture vessel and grow. Second, MSCs should have CD73, CD90, and CD105 as cell surface antigens. Also, CD11b, CD14, CD19, CD34, CD45, CD79α, and HLA-DR antigens, which are hematopoietic stem cell antigens, should not exist on MSCs. Third, MSCs must be able to differentiate into osteoblasts, adipocytes, and chondrocytes in vitro [4]. After the discovery of bone marrow-derived MSCs (BM-MSCs), several other MSC sources have been reported, including endometrium [5], dental pulp tissues [6], skeletal muscles [7], placenta [8], adipose tissue [9], umbilical cord blood [10], and Wharton’s jelly [11] are sources of MSCs. MSCs are suitable for cell therapy because: (a) They have stemness potency; b) They are easy to isolate from original tissues; (c) They have less severe ethical issues as compared to embryonic stem cells (ESC); (d) Unlike induced pluripotent stem cells (iPSC), they carry a lower risk of teratoma-formation [12,13]; and (e) They are useful for a variety of therapeutic applications because of their ability to migrate to damaged tissue by chemoattraction [14]. Hence, it is possible to apply MSCs for the treatment of tissues of different origins [15,16,17,18,19].

The skin is continuously exposed to a variety of injuries. In dermatology, MSCs have demonstrated the potential for skin regeneration in many reported cases [20,21]. Additionally, due to the modern population’s increased esthetic standards, the interest in keeping a youthful appearance has also increased. Therefore, skin rejuvenation using MSCs is a treatment that attracts attention [22,23,24]. This review focuses on recent applications of MSCs and MSC-derived appendages in skin regeneration and rejuvenation.

## 2. Skin Structure

**Figure 1:**
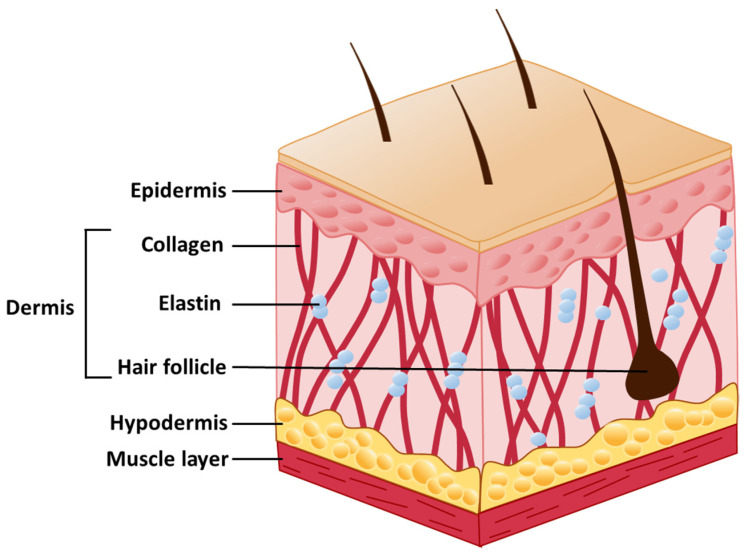
Schematic representation of the human skin structure.

The various layers of the skin have distinct structures and functions that work together to protect internal organs and serve diverse biological functions. The skin is composed of three major layers: epidermis, dermis, and hypodermis (Figure 1).

The epidermis, the outermost layer, plays a major defensive role [25]. This layer protects the skin from damage and stress, while also limiting the passage of water and chemical absorption [26]. It is constituted predominantly of keratinocytes, which are present in the epidermis in different maturation states, constituting around 95% of the layer [27]. These cells produce multiple keratins, which are major structural proteins that provide strength to the skin [28]. The epidermis is subdivided into five distinct strata: stratum corneum, stratum lucidum, stratum granulosum, stratum spinosum, and stratum basale [29]. Keratinocyte stem cells are located in the basal layer and gradually differentiate across the layers until they become terminally differentiated in the stratum corneum, being gradually replaced by keratinocytes prevenient from the bottom layers [30,31]. In the stratum spinosum, Langerhans cells are involved in an immune response, and protect the skin from microbial agents [32]. Additionally, Merkel cells function as sensorial receptors for stimuli, including pain, temperature, and touch [33]. Melanocytes are also present, producing melanin and transferring it to keratinocytes. Melanin provides pigment to the skin and hair, and also protects the skin from damage by ultraviolet (UV) radiation [34,35].

Between the epidermis and dermis, there is a cutaneous basement membrane zone, which connects basal keratinocytes with collagen fibers located on the surface of the dermis [36]. The main function of this structure is to provide adhesion between both layers, Since the epidermis is avascular, the basement membrane zone allows oxygen and nutrient exchange from the vascular dermis to the epidermis [37].

The dermis plays a crucial role in cushioning the body and providing structure. This layer is arranged as a mesh-like network consisting of connective tissue, blood vessels, lymph vessels, and mast cells [38]. Connective tissue is mainly formed by fibroblasts, which are responsible for the synthesis of elastin and collagen proteins [39]. Elastin proteins play a role in assuring elasticity and resilience to the skin. Collagen fibers are structural proteins that play important roles in stretching and providing tensile strength to the skin [40]. Mast cells are responsible for the inflammatory response of the skin to combat microorganisms, allergens, and physical injury [41].

The hypodermis is the deepest layer of the skin, being mainly composed of adipocytes. A rich vascular plexus extends from this layer to the dermis, supplying it with blood. Furthermore, the hypodermis makes the connection of the skin to muscles. Deep wounds that reach the hypodermis, causing its loss or damage, constitute complicated cases for wound healing [42,43].

Human skin and rat skin differ in histological, phenotypic, immunological and molecular domains. Therefore, given that rodents are models more accessible for investigation, we should consider their differences when studying wound healing. Firstly, even though the epidermis, dermis and hypodermis are structurally similar in humans and mice, their thicknesses are distinct. Human skin is usually 5 to 10 layers of epidermis and over 100 μm of thickness, but mice skin is 2 to 3 layers of epidermis and less than 25 μm thick. As a result, mice skin has a lower barrier function and increased absorption than humans skin [44]. Furthermore, male mice dermis is 40% thicker than the female mice dermis. Furthermore, contrarily to humans, mice skin possesses panniculus carnosus, a thin muscle layer that gives the skin contraction properties. This muscle is responsible for almost 90% of the wound of mice closure process [45]. On the other hand, human skin wound closure depends on reepithelization and granulation tissue formations In research, using mice as skin models should take these different factors into account for experimental design and result interpretation. Additionally, concerning wound healing, inflammatory reactions control the healing capacity. Humans and mice have different percentages of leukocytes, 10–25% neutrophils and 75–90% lymphocytes in mice, and 50–70% and 30–50% respectively, in humans [46]. Despite that, the effects of these differences in wound healing are not clear. In addition, human skin neutrophils express defensin, an antimicrobial peptide that aids in the case of infection, but mice skin does not. [45,47,48]. Comparing to mice, several other mammals are physiologically more close to humans [49]. For example, the structure of skin and wound healing mechanisms of pigs are similar to humans. However, pigs are not as well researched physiologically as mice, since the cost of maintaining pigs in a lab is higher, and surgical operation is more complicated [49]. Considering these limitations, mice are more widely used for skin research [49].

## 3. Applications of Mesenchymal Stem Cells in Skin Regeneration

### 3.1. Wound Healing

Wounds can be divided into acute and chronic, depending on the time and progress of the healing process. Additionally, post-infection and post-inflammatory wounds are also significant problems. Thus, it is essential to develop technologies to aid against skin loss due to wounds.

**Figure 2:**
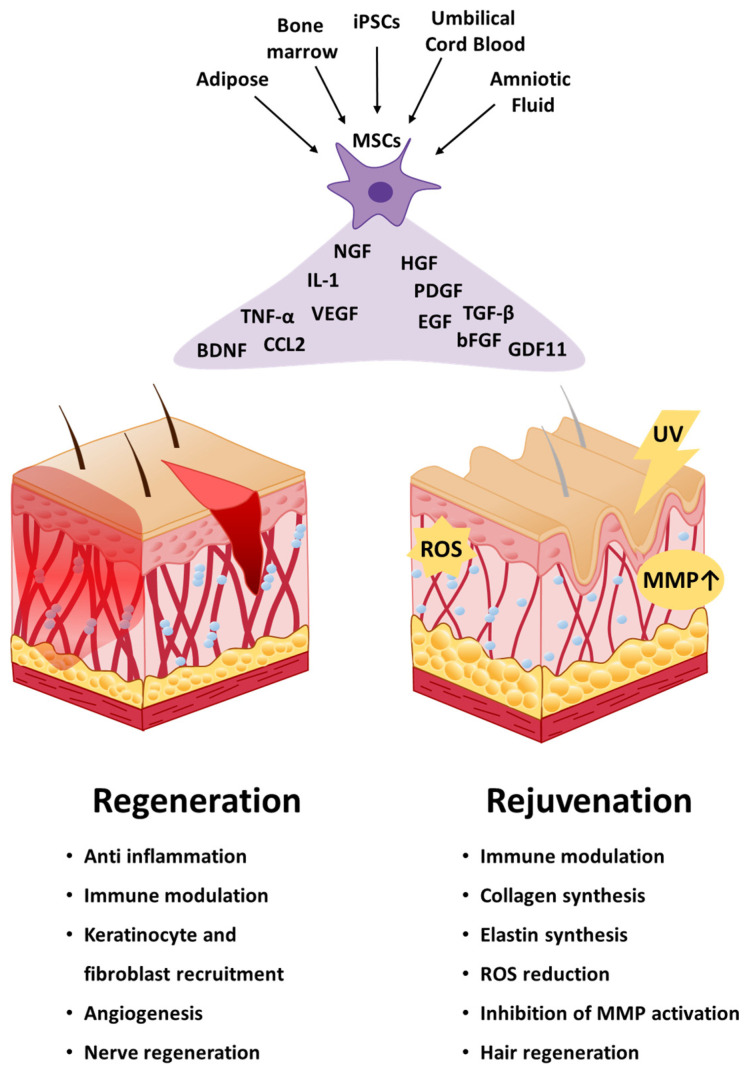
MSCs healing mechanism in skin regeneration and rejuvenation.

Typically, skin wound healing comprises four overlapping phases: hemostasis (coagulation), inflammation (infiltration of mononuclear cells), proliferation (epithelization, fibroplasia, angiogenesis) and maturation (collagen deposit, formation of scaring tissue) [50]. MSCs aid in all phases of the wound healing process. Application of MSCs for skin therapy can enhance wound healing and curtail scarring. MSCs migrate to the spot of skin injury, inhibit inflammation, and elevate the proliferation and differentiation potential of fibroblasts, epidermal cells, and endothelial cells (Figure 2) [51,52]. Recent studies have reported that MSC-derived cultured media (MSCs-CM), extracellular matrix (ECM), exosome, platelet-rich plasma (PRP), and cytokines treat injuries in diverse tissue types (Table 1).

The inflammatory phase is important for the wound healing process, as it leads to the recruitment of immune cells to reduce pathogens and clear the injury. However, chronic inflammation can postpone skin healing. MSCs can inhibit inflammatory responses in several ways. Chiossone et al. (2016) showed that MSCs promote polarization of macrophages to an M2-like phenotype, a type of macrophage that reduces inflammation and immunosuppressive function [53]. Moreover, the MSC-induced M2-like phenotype macrophages (M^MSC^) interact with natural killer (NK) cells and inhibit the expression of NK activation-related proteins such as NKp44, CD25, CD69, and interferon-gamma (IFN-γ). Furthermore, M^MSC^ can inhibit T cell proliferation by promoting the multiplication of Tregs [53]. Luz-Crawford et al. (2016) provided evidence of the critical role of interleukin-1 receptor antagonist (IL1RA) secreted by MSCs in inducing M^MSC^ and inhibiting B cell maturation in an IL1RA knock-in mouse model [74]. Interleukin-1 (IL-1) is known to accelerate T-helper 17 (Th17) cell differentiation [75]. Th17 cells express IL-17, which is a marker of inflammatory cytokines in many tissues. Therefore, IL1RA decreases the differentiation of Th17 cells and causes an increase in the anti-inflammatory effect of the cells. Zhao et al. (2013) revealed that IL1RA from BM-MSCs inhibits the production and activity of IL-1 and TNF-α, which are pro-inflammatory cytokines [76]. These studies indicate that MSCs have anti-inflammatory ability through modulation of macrophage polarization and expression of anti-inflammatory cytokines (Figure 2).

In the proliferative phase, MSCs manipulate macrophages to recruit keratinocytes and fibroblasts (Figure 2). Macrophages release epidermal growth factor (EGF) and transforming growth factor-α (TGF-α) to stimulate the migration and proliferation of keratinocytes [77]. Fibroblasts increase the migration and proliferation of keratinocytes via EGF, fibronectin, and keratinocyte growth factor (KGF) [77]. Keratinocytes also stimulate fibroblasts by expressing fibronectin, laminin 332, and tenascin [77]. Li et al. (2015) showed that high glucose and lipopolysaccharide inhibits the migration and proliferation of rat keratinocytes [78]. Furthermore, MSCs-CM can stimulate the migration and proliferation of keratinocytes [78]. Smith et al. (2010) revealed that BM-MSCs release soluble signaling factors that increase migration, proliferation, and chemotaxis of dermal fibroblasts [79]. MSCs can lead to angiogenesis at the site of the wound (Figure 2). Rustad et al. (2012) showed that MSCs-CM within hydrogels increased VEGF expression levels and resulted in faster wound healing than an injection of only MSCs into the wounded skin area [68]. Furthermore, Qiu et al. (2020) noted that MSCs educated by exposure to exosomes from neonatal mouse serum significantly improved wound healing [80]. Moreover, they found that the exosomes of educated MSCs significantly increased wound healing by inducing angiogenesis [80]. Martin-Piedra et al. (2019) used AD-MSCs, dental pulp-derived MSCs (DP-MSCs), Wharton’s jelly-derived MSCs, and BM-MSCs for epidermal regeneration, by tissue engineering and surgical grafting in animal models. The study illustrated that the partial epithelial differentiation ability of these cells could be used to generate bioengineered human skin substitutes for epidermal repair [81].

Furthermore, another important aspect involved in skin wound healing is the recovery of nerve function [69]. Skin wound healing aims to recover the protective ability of skin, and restore neuronal excitation functions through nerve regeneration. First, endogenous MSCs migrate toward the injury site because of chemoattractants. Stromal cell-derived factor-1 (SDF-1) is a well-investigated chemoattractant for the recruitment of MSCs. In the second stage, MSCs promote neuronal regeneration. bFGF, nerve growth factor (NGF), and brain-derived neurotrophic factor (BDNF) are important secretory factors that promote nerve regeneration (Figure 2) [82].

The ECM exists within all tissues and organs and contributes crucially to physical scaffolding for the cellular construction and initiation of signaling bioactive factors [83]. ECM is composed of proteins, polysaccharides and water, but each tissue has an unique composition and topology [83]. Collagen, a component of the ECM of skin, facilitates the migration of keratinocytes to reconstruct the damaged epidermis, as collagen-based materials also improve wound healing [84,85]. Zhou et al. (2019) showed that a combination of AD-MSCs and their ECM increases wound healing [86]. Platelet-rich plasma (PRP) is a rich source of cytokines and growth factors important for wound healing, including EGF, bFGF, HGF, PDGF, TGF-β1 and VEGF [87]. Recent studies have shown that PRP has an anti-inflammatory effect and regulates macrophages to increase wound healing [87]. Hersant et al. (2019) showed that a treatment combining PRP and MSCs improves mouse wound closure and proangiogenic properties in wound sites [88]. Holmes et al. (2018) studied a treatment mixture of leukocyte-high PRP and bone marrow concentrate to induce the recruitment BM-MSCs in the microfluidic device [62]. Moreover, Paganelli et al. (2019) used MSCs derived from adipose tissue to build a dermal substitute for wound healing, with high biocompatibility and good mechanical properties [89]. In addition, Zhang et al. (2018) revealed that AD-MSCs increase wound healing via their paracrine function [90]. They showed that AD-MSC-derived exosomes improve wound healing by regulating the proliferation and migration of fibroblasts, and optimizing collagen deposition [90]. Furthermore, AD-MSC-derived conditioned media increased the migration of skin fibroblasts and elevated wound healing in vivo.

These reports illustrate that MSCs and MSC-derived cytokines, exosomes, ECM, PRP, and CM have wound healing capacity. Various cell-derived MSCs and their derived molecules have applications in wound healing (Table 1). These applications can be subjected to clinical trials, and optimized treatment plans and patient types can be decided.

### 3.2. Burn Injury

Burns are one of the main injuries worldwide [50]. Burn injuries are classified as first to third-degree burns (1~3°). The recovery depends on the severity of the burn, and two weeks are needed for recovery from superficial burning and minimal scarring. Severe burn injury includes third-degree (3°) burns and damage to the full thickness of the skin [91]. Angiogenesis is vital for the blood supply required to heal severe burn injuries [92].

Recently, many studies have reported that MSCs aid in healing burn injuries (Figure 2) [93,94,95,96]. MSCs increase wound closure and angiogenesis, and minimize scarring. BM-MSCs induced burn healing in a rat model by the expression of collagen 1 and integrin α2β1 [97]. In the same burn injury rat model, umbilical cord-derived MSCs (UC-MSCs) promoted burn healing through an immunosuppressive effect [98]. However, the healing mechanism of burn injuries by MSCs is not fully understood yet. Additionally, the attachment of transplanted MSCs to wounds is limited. The rate of engraftment of MSCs into organs is less than 3%, as reported in heart [99], kidney [100], liver [101] and pancreatic [102] injury models [20]. Because of this poor engraftment of MSCs, detailed studies are needed to increase the probability of engraftment of MSCs in damaged skin. There are two methods to inject MSCs into the body: Firstly, MSCs can be delivered into the tissue locally, by diverse scaffolds embedding MSCs. Several scaffolds methods have been developed to help in the transplant of MSC for tissue engineering clinical therapy, being composed of biodegradable, ceramic, matrix, synthetic, or alternative materials [103]. Secondly, MSCs can be injected by intracardiac, intramuscular, or intraperitoneal injections. It is also possible to inject via intravascular injection, either by arterial (IA) or venous (IV) injections. Relevantly, Krean et al. (2013) showed that MSCs injection by IA is more effectively spread than IV injections [104]. By IV injection into the tail vein, MSCs clearly capture in the lungs, however, when MSCs were delivered by IA injection through the aortic arch, the cells were more equally spread in the entire animal body [104]. These two methods for injecting MSCs into the body should be more developed for increasing the rate of engraftment of MSCs engagement into the skin.

## 4. Applications of Mesenchymal Stem Cells in Skin Rejuvenation

### 4.1. Antiaging

Aged skin is highly associated with an unpleasing esthetic, which occurs due to loss of function and structural degeneration of the skin [105]. This can result in more serious complications, including more susceptibility to diseases such as eczema, dermatitis, autoimmune disorders, and melanoma [106]. With aging, the skin naturally loses its collagen content and elastic fibers become deranged [107]. Additionally, aged skin demonstrates an increase in oxidant activity [108], and an increase in the production of matrix metalloproteases (MMP), which are typically involved in matrix degradation. Additionally, exposure to UV light is known to promote premature aging of the skin, namely photoaging (Figure 2) [109]. Thus, rejuvenation therapies, which focus on the prevention and reversal of skin aging are in high demand in our society, which increasingly aims to maintain a youthful appearance and improve their health.

AD-MSCs have been gaining attention in skin antiaging therapy because of their efficient re-epithelization and secretion of several growth factors necessary for skin regeneration [24]. In recent years, Charles-de-Sá et al. (2015) observed the histological and structural modifications in aged facial skin after the injection of expanded AD-MSCs, collected from fat removed by liposuction [110]. Treatment with AD-MSCs caused an increase in elastic fibers in the superficial layer of the dermis and modified the collagen and reticular fiber networks, which became more arranged. Subsequently, AD-MSCs were observed to induce complete regeneration of solar elastosis in photoaged skin [111]. The transplantation of AD-MSCs leads to complete regeneration of dermal elastic matrix components, including oxytalan, elaunin, and elastin fibrillary networks. In solar-aged skin, the normal elastin matrix is usually lost, and AD-MSC-mediated treatment successfully reversed the inhibition of precursor molecules involved in neoelastinogenesis. This was observed by their high immunoreactivity, which indicated a high de novo formation. Additionally, the elastotic abnormal elastin deposits in the deeper dermal layers were degraded and replaced by typically polymerized elastic fiber networks. This was hypothesized to have been caused by the activation of cathepsin K, which allows reparative and hyperplastic processes after sun exposure.

Another way to use AD-MSCs in antiaging therapy, in a “cell-free” method of treatment, is by using extracellular vesicles (EVs), which have several advantages over stem cells and their safety issues. Adipose-derived mesenchymal stem cells extracellular vesicles (AD-MSCs-EVs) have anti-photoaging potential and were analyzed as subcutaneous injections in photoaged mice models [112]. The treatment resulted in a decrease in skin wrinkles and promotion of epidermal cell proliferation. Additionally, macrophage infiltration and reactive oxygen species (ROS) production were reduced, which inhibited MMP activation and collagen degradation (Figure 2). Moreover, in vitro analysis showed increased fibroblast activity and protection from UVB-induced senescence.

Amniotic membrane-derived mesenchymal stem cells (AM-MSCs) have also gained popularity as agents for improving photoaging, due to their abundance, easy acquisition, growth factors, and cytokines. A study conducted in 2019 by Prakoeswa et al. used AM-MSC-conditioned medium to treat photoaged human patients, and microneedling was used to enhance penetration of the medium [113]. Clinical photoaging (pore, wrinkle, spot polarization, spot UV, and skin tone) improved in the treatment groups, as observed by the surface skin analysis system, Janus. AM-MSCs were predicted to improve proliferation and migration of dermal fibroblasts and epidermal keratinocytes, and increase collagen synthesis.

Furthermore, BM-MSCs have recently been observed to have beneficial effects on skin aging. In a study conducted by Liu et al. in 2017, the effects of BM-MSCs on skin aging were analyzed on mice models subjected to D-galactose-induced aging [114]. D-galactose is a monosaccharide sugar that is known to cause mitochondrial dysfunction and oxidative stress in cells. Treatment with BM-MSCs resulted in reduced antioxidant activity, as observed by a reduced content of malondialdehyde (MDA), which is formed by the degradation of polyunsaturated lipids by ROS, causing peroxidative tissue damage. Furthermore, superoxide dismutase (SOD) activity increased, demonstrating an improved dismutation of superoxide radicals to hydrogen peroxide and oxygen. Finally, the glutathione-peroxidase (GSP-Px) content also increased, leading to a better reduction of hydrogen peroxide to water, thus preventing lipid peroxidation.

Human umbilical cord blood-derived mesenchymal stem cells (UCB-MSCs) are known for their rapid proliferation and immunomodulatory capacity, while also being easy to isolate, in contrast to typical adult MSCs (Figure 2). These cells have also been a target for antiaging studies on skin. Kim et al. (2018) found that a conditioned medium of UCB-MSCs contained several growth factors such as EGF, bFGF, TGF-β, PDGF, hepatocyte growth factor (HGF), collagen type 1, and a rejuvenation factor called growth differentiation factor 11 (GDF-11) (Figure 2) [115]. Furthermore, a cream based on UCB-MSC-conditioned medium was used in vivo, and its effects on dermal density and wrinkles in human patients were analyzed. After daily treatment for four weeks, evaluation with digital micromirror devices demonstrated that skin density improved by 2.46% and eye-end wrinkles decreased. Another approach for skin rejuvenation is the use of EVs derived from UC-MSCs [116]. Engineered EVs (eEVs) were obtained using ultrasonication, and showed functions similar to those of naturally secreted EVs. Comparative tests demonstrated that eEVs promoted fibroblast proliferation and migration in vitro. They also increased the expression of proteins involved in the maintenance of the extracellular matrix, such as collagen, elastin, and fibronectin, and inhibited the expression of MMP-1 and MMP-3.

### 4.2. Hair Loss

Androgenic alopecia is a form of hair loss that can occur in both men and women. In men, this condition is also referred to as male-pattern baldness. Male pattern hair loss converts testosterone in hair follicle cells into a more potent metabolite, dihydrotestosterone (DHT). DHT binds to the androgen receptor in the hair follicle, thereby lowering the cyclic AMP (cAMP) concentration in the cell. It reduces sugar metabolism in hair follicles and suppresses energy supply to shorten the hair follicle growth period. As a consequence, the duration of the resting phase of the hair increases, and the hair follicle gradually becomes thinner and shorter [117,118].

The role of stem cells located in the hair follicle bulge is vital for hair regeneration, involving the Wnt/β-catenin cycle [119]. The dermal papilla is essential for hair growth and hair loss occurs when the dermal papilla is inhibited from secreting growth factors [120,121]. Huang et al. (2016) investigated the interactions of dermal papilla cells with AD-MSCs in increasing hair formation [122]. Another study showed that when human amniotic fluid-MSCs-CM (AF-MSCs-CM) were injected subcutaneously around a full-thickness wound in rats, wound healing was facilitated and hair regrowth was observed at the wound site [123].

Similarly, there are reports that BM-MSCs play a role in wound repair and improve hair regrowth [124]. Dong et al. (2014) reported that over-expression of Wnt1a by BM-MSCs-CM stimulated the induction ability of mouse dermal papilla cells. Thus, BM-MSCs promote progression of hair cycle and lead to hair regeneration [123]. Park et al. (2019) showed that overexpression of Nanog by AF-MSCs promotes activity of dermal papilla cells and increases hair follicle recycling [125]. Rajendran et al. (2017) reported that mouse BM-MSCs-EVs stimulate proliferation and migration of dermal papilla cells. Fluorescence monitoring confirmed the uptake of BM-MSCs-EVs in dermal papilla cells which lead to the anagen stage of hair growth in a mouse model [126]. These results reveal that MSCs and MSC-derived appendages could be candidates for hair regrowth stimuli and hair loss treatment (Figure 2).

## 5. Induced Pluripotent Stem Cell-derived Mesenchymal Stem Cells

Typically, MSCs are harvested from adult adipose tissue, bone marrow, or umbilical cord. However, MSCs can be obtained from only approximately one-third of the umbilical cord blood. Furthermore, only 0.001–0.01% of the bone marrow cells allow harvesting of MSCs. Also, only 0.05% of adipose tissue from a donor can be used as a source of MSCs [127,128]. For the clinical application of MSCs into the human body, MSCs cell needs 1–3 × 10^6^ cells/kg body weight [129,130,131,132,133]. The methods for MSCs harvesting are considered painful and difficult procedures, and require patient permission [134]. Furthermore, as mentioned above, the limited number of cells obtained from tissues is an issue. Additionally, restrained in vitro proliferation capacity is another difficulty in obtaining uniform populations for clinical trials. Since iPSCs are generated by somatic cells previously obtained from a patient, iPSCs are an easier and more ethical approach, comparing to MSCs concerning biopsy [135].

**Figure 3:**
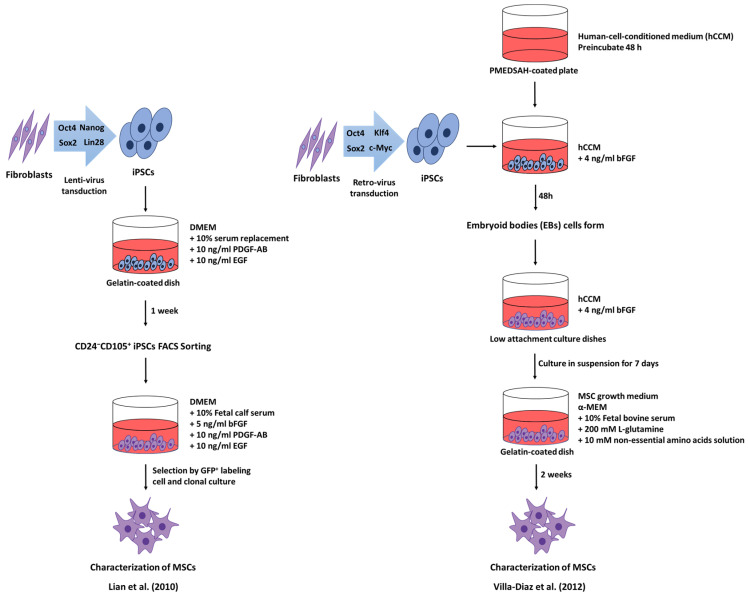
Methodology for obtention of induced pluripotent stem cell (iPSCs)-derived MSCs.

Takahashi et al. (2007) investigated the generation of iPSCs from adult human dermal fibroblasts by Yamanaka factors (Oct3/4, Sox2, Klf4, and c-Myc) through retroviral transduction [136]. Recent studies have shown that human iPSC-derived MSCs (iMSCs) are capable of aiding in various diseases. In a study by Lian et al. (2010) [137], MSCs were generated from iPSCs, with features similar to human BM-MSCs in terms of marker expression and differentiation potential (Figure 3). First, iPSCs were generated from IMR90 fibroblast cells by transduction of Oct4, Nanog, Sox2, and Lin28 factors through lentivirus transduction. To differentiate human iPSCs into MSCs, the authors used a published clinical protocol. iPSCs were placed on a gelatin-coated dish containing Dulbecco’s modified Eagle’s medium (DMEM) supplemented with 10% serum replacement (SR), 10 ng/mL bFGF, 10 ng/mL PDGF-AB, and 10 ng/mL EGF, to promote the proliferation of MSCs. After 1 week, differentiated CD24^−^CD105^+^ iPSCs were harvested by fluorescence-activated cell sorting (FACS). The CD24^−^CD105^+^ cells were cultured in DMEM with 10% fetal calf serum, 5 ng/mL bFGF, 10 ng/mL PDGF-AB, and 10 ng/mL EGF. Adult human BM-MSCs were used as a control for the comparable characteristics of MSCs [137]. By transplantation of iMSCs into mice with severe hindlimb ischemia, the symptoms were reduced due to muscle regeneration and angiogenesis induced by iMSCs. Additionally, tests revealed that iMSCs and BM-MSCs have different capabilities in attenuating severe hindlimb ischemia by muscle regeneration, angiogenesis, and paracrine factor secretion. This result indicates that iMSCs have a better therapeutic capacity than BM-MSCs [137]. Additionally, Xu et al. (2019) revealed that iMSCs have different functions and gene expression patterns than BM-MSCs [138]. It was observed that the expression of CD73, CD90, and CD105, markers of MSCs, was significantly higher in iMSCs than in BM-MSCs. Further, iMSCs showed an increased expression of both KDR and MSX2 mRNA, as compared to BM-MSCs. Furthermore, BM-MSCs had a relatively high PDGFRα mRNA expression. These results suggest that iMSCs are distinguished from primary MSCs, and that iMSCs can be used in treatment methods which are beyond the limitations of primary MSCs [138].

Many experiments were attempted to differentiate iPSCs into MSCs. For example, Villa-Diaz et al. (2012) demonstrated that iPSCs cultured on synthetic substrates differentiated into MSCs (Figure 3) [139]. iPSCs were placed on poly [2-(methacryloyloxy)ethyl dimethyl-(3-sulfopropyl)ammonium hydroxide (PMEDSAH)-coated plates with human-cell-conditioned medium (hCCM) supplemented with 4 ng/mL bFGF2. PMEDSAH-coated plates were preincubated with hCCM for at least 48 h at 37 °C in a 5% CO_2_ incubator. Embryoid bodies (EBs) were formed, and cultured in suspension for 7 d with hCCM. Almost 70 EBs were cultured on 0.1% gelatin-coated dishes in α-minimum essential medium (α-MEM) with 10% fetal bovine serum (FBS), 200 mM L-glutamine, and 10 mM non-essential amino acids solution (NEAA), to promote the differentiation of iPSCs into MSCs. EBs were cultured for 2 weeks until the cells had a homogeneous fibroblastic morphology in the culture dish. The results showed that iMSCs were successfully differentiated, and had functional capacity, especially of bone formation in vivo [139]. In another method, SB-431542, a TGF-β inhibitor, was used to promote the differentiation of iPSCs into MSCs. iMSCs generated after 10 days of SB-431542 treatment revealed characteristics of MSCs such as differentiation potential and immunophenotype [140]. Several methods are currently being tried to obtain functionally useful iMSCs.

Recent studies demonstrate that iMSCs immune modulation and teratoma formation properties compare with MSCs or iPSCs. Soontararak et al. (2018) demonstrated that iMSCs have equal healing potential as AD-MSCs in mouse inflammatory bowel disease models [141]. Fu et al. (2012) and Gao et al. (2017) show that iMSCs modulate T-cell and dendritic cell function similarly to AD-MSCs or BM-MSCs [142,143]. Chow et al. (2017) discovered that iPSCs could lead to the formation of teratomas after 20 days of subcutaneous injection into immune-deficient mice. However, iMSCs intravascularly injected into dog models did not lead to the formation of tumors [144]. Wei et al. (2012) found that iMSCs did not result in teratoma formation in SCID mice models [145]. These results indicate that iMSCs could be a useful clinical therapy by immune modulation and safe for tumor formation rather than iPSCs.

Recently, several studies have been using iMSCs for skin regeneration and skin rejuvenation. Nakayama et al. (2018) successfully differentiated keratinocyte-derived iPSCs (KC-iPSCs). This group received keratinocytes from patients with human recessive dystrophic epidermolysis bullosa (RDEB) and obtained KC-iPSC-derived MSCs (KC-iMSCs) [71]. KC-iMSCs were injected subcutaneously and intravenously into immunodeficient mice with skin injury. After transplantation, human collagen VII was found at the dermal-epidermal junction, indicating successful wound healing. In addition, Kim et al. (2018) revealed that exosomes secreted by iMSCs (iMSCs-exo) increase the proliferation of human keratinocytes and dermal fibroblasts [146]. Also, according to a study by Veraitch et al. (2017), iMSCs improved the properties of dermal papilla cells and contributed to increasing the hair-like structure morphology in an immunodeficient mouse model [147]. Furthermore, Spitzhorn et al. (2019) reported that human iMSCs express genes related to rejuvenation. In this study, iPSCs were used to obtained induced fetal femur-derived MSCs and adult BM-MSCs. Both types of iMSCs had common MSC cell surface markers and expressed rejuvenation-related genes such as CDKN1C, DNMT3B, GCNT2, INHBE, and POU5F1P1. These results suggest that iMSCs can acquire rejuvenation-related genes regardless of donor age and MSC source. The iMSCs concept avoids the shortcomings associated with the use of adult MSCs. Therefore, iMSCs may prove useful for future applications in various clinical settings [148].

## 6. Embryonic Stem Cells-Derived Mesenchymal Stem Cells

Evans et al. (1981) first discovered embryonic stem cells (ESCs), originated from the inner cell mass of mouse blastocysts and, Thomson et al. (1998), was the first to report studies with human ESCs. These cells have the capacity to differentiate into all three germ layers (mesoderm, endoderm, and ectoderm). ESCs are considered to be able to overcome the limitations of adult stems cells, however, for clinical application, ESCs have higher risk of tumorigenicity, comparing to iPSCs, and the possibility of immune rejection. The major limitation of the development of ESC-based clinical therapies is the sacrifice of an embryo [135], which constitutes a major ethical issue. If overcome properly, ESC-derived MSCs (eMSCs) based clinical trials could be considered for skin regeneration and rejuvenation medicine.

Barberi et al. (2005) reported the first example of differentiation of ESCs into MSCs [149] and, over the years, research with eMSCs has been expanding [150,151,152]. Hwang et al. (2008) reported that transplanted eMSCs into the knee joint cartilage defect area promoted cartilage repair [153]. Furthermore, Laurila et al. (2009) transplanted eMSCs and BM-MSCs into rat ischemic model and revealed that the eMSCs and BM-MSCs have a similar capacity to enhance angiogenesis and cell proliferation due to secretion of growth factors [154]. Clinical application for skin regeneration and rejuvenation by eMSCs is still scarce, but former publications indicate that eMSC can be applied to regenerative medicine [155,156]. Yoon et al. (2018) first published evidence that eMSCs promote wound healing in pressure ulcers. It was demonstrated that eMSCs increase wound closure, vessel formation and expression of collagen type I and III, fibronectin, and fibroblast-specific protein-1 (FSP-1) [72].

## 7. Conclusions

Novel studies on MSCs have demonstrated their potential in skin therapy. Transplantation of MSCs is considered a powerful tool for regeneration and rejuvenation of skin, as MSCs are a promising source of skin cells. Recent advances have revealed that MSCs have many benefits in treating the skin. For example, research on MSCs demonstrates efficacy in healing, especially due to the improvement of immune function by macrophage activation and cytokine production. Furthermore, MSCs have been shown to improve skin conditions by ameliorating antioxidant activity, promoting cell proliferation, and improving overall skin morphology. However, further studies focusing on the underlying molecular mechanisms are still necessary to guarantee the safe implementation of these methods. Despite all the advantages and benefits of MSC therapy, there are still obstacles such as their low frequency in tissues and the limited proliferative potential of MSCs derived from adult sources. Thus, we propose iMSCs as a promising target for skin therapy research. Despite being relatively new, this technology has demonstrated great potential in stem cell research, considering the high self-renewal capacity and differentiation ability of iMSCs.

## Figures and Tables

**Table 1:** Applications of mesenchymal stem cells (MSCs) in wound healing.

Wound Healing Process	Treatment to MSCs	Function of MSCs	Source of MSCs	Model	Reference
Anti-inflammation	-	Polarization of macrophages to an M2 phenotype	BM	MSCs co-culture with macrophage	[53]
	MSC-derived exosome	Polarization of macrophages to an M2 phenotype	BM	MSCs co-culture with macrophage	[54]
	TNF-α, IL-6	Polarization of macrophages to an M2 phenotype	Gingiva	MSCs co-culture with macrophage	[15]
	TSG-6	Polarization of macrophages to an M2 phenotype	BM	Diabetic mice model	[55]
	TNF-α	Limiting macrophage activation	BM	Skin injury mice model	[56]
	siTSG-6 (negative effect)	Polarization of macrophages to an M2 phenotype	cAD	Inflammatory bowel disease mice model	[57]
Proliferation	CXCR4 antagonist (negative effect)	Chemotaxis of MSCs	BM	Burn mice model	[58]
	PRP	Chemotaxis of MSCs	AF	Transwell migration assay	[59]
	PRP	Fibroblast migration	AD	Wound healing assay in culture dish	[60]
	PRP	Re-epithelialization	AD	Skin injury mice model	[61]
	PRP	Chemotaxis of MSCs	BM	Chemotaxis device	[62]
	EMPB	Migration of MSCs	Endogenous MSCs in mice	Diabetic mice model	[63]
	Cinnamtannin B-1	Migration of MSCs	Endogenous MSCs in mice	Diabetic mice model	[64]
Angiogenesis	Low-level laser therapy	VEGF, bFGF secretion in the wound bed	cAD	Skin injury mice model	[65]
	-	CCL2	Primary MSCs in CCL2-KO mice	Skin injury mice model	[66]
	Negative pressure wound therapy	CD31, VEGF, α-SMA	BM	Skin injury mice model	[67]
	Biomimetic hydrogel scaffold	Wound vascularization	BM	Skin injury mice model	[68]
Increase in wound closure	Self-adaptive all-in-one delivery chip	Skin nerve regeneration	BM	Skin injury mice model	[69]
	Chitin nanofiber-based hydrogel	Granulation tissue formation	BM	Skin injury mice model	[70]
	-	Collagen type VII	iPSC	Skin injury mice model	[71]
	CTGF	Fibroblast differentiation	ESCs	Skin pressure ulcer mice model	[72]
	ECM	VEGF, PDGF, EGF	UCB	Diabetic rat model	[73]

BM: bone marrow; cAD: canine adipose tissue; AD: adipose tissue; iPSC: induced pluripotent stem cells; AF: amniotic fluid; UCB: Umbilical cord blood, VEGF: vascular endothelial growth factor; bFGF: basic fibroblast growth factor; CCL2: chemokine (C-C motif) ligand 2; TNF-α: tumor necrosis factor-α; TSG-6: tumor necrosis factor-α–stimulated gene/protein-6; siTSG-6; CTGF: connective tissue growth factor; PDGF: platelet-derived growth factor; EGF: epidermal growth factor; TSG-6 siRNA transfection; KO: knockout; PRP: Platelet-rich plasma; EMPB: ethanol extract from *Mallotus philippinensis*, a plant in the spurge family, bark.

## Data Availability

Not applicable.
